# P-1364. Avoidance of Fluoroquinolones in Patients with Isoniazid-Monoresistant Tuberculosis in a High Income Setting

**DOI:** 10.1093/ofid/ofaf695.1551

**Published:** 2026-01-11

**Authors:** Anna Daunt, Sadia Qureshi, Denis Keane, Ashley Whittington

**Affiliations:** London North West University Healthcare NHS Trust, London, England, United Kingdom; London North West University Healthcare Trust, London, England, United Kingdom; London North West University Healthcare NHS Trust, London, England, United Kingdom; London North West University Healthcare NHS Trust, London, England, United Kingdom

## Abstract

**Background:**

For patients with isoniazid mono-resistant tuberculosis (TB), evidence surrounding optimal regimen is lacking. WHO (World Health Organisation) recommend 6 months of quadruple therapy including a fluoroquinolone. This is based on individual patient data from 251 quinolone treated patients and is susceptible to confounding. In the UK, National Institute for Health and Care Excellence (NICE) guidelines caution against quinolone use unless unavoidable, recommending 2 months of rifampicin, pyrazinamide and ethambutol, followed by 7 months rifampicin and ethambutol. This regimen has never been directly compared with a quinolone containing regimen.

**Table 1:**
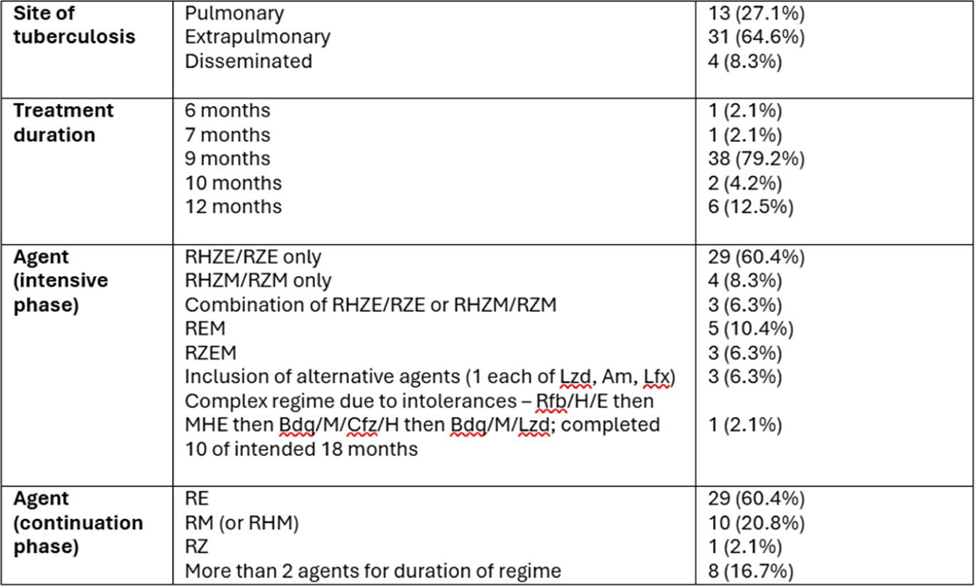
details of cohort and treatment regimen for patients for whom complete medical records were available (n=48) Abbreviations: Am; amikacin; Bdq; bedaquiline; Cfz; clofazamine; E; ethambutol; H; isoniazid; Lfx; levofloxacin; Lzd; linezolid M; moxifloxacin; R; rifampicin; Rfb; rifabutin; Z; pyrazinamide

**Methods:**

Retrospective review of medical records of all patients treated for isoniazid-resistant TB at London North West University Healthcare NHS Trust from 2018 to present. The UK National TB Surveillance database (NTBS) was used to identify patients and relapsed infection.

**Results:**

91 patients were registered as isoniazid-resistant on NTBS. 19 had additional antibiotic resistance precluding use of WHO and NICE regimens. 60/72 patients had sufficient clinical records available to determine regimen. 10 remain on treatment and 2 have been lost to follow up. 48 patients had full regime and outcome data available. Of these, 21 (43.8%) followed NICE regimen exactly. No patients followed the WHO regimen. 20 (41.2%) received quinolones at any point in their regime, usually due ethambutol contraindication or higher burden of disease. Further cohort and regimen details are available in Table 1. Treatment was successfully completed for 47/48 (97.9%) patients with complete clinical records. 1 patient, who completed 10 of a planned 18 months treatment, was re-referred to TB services due to an abnormal chest X-ray, but did not attend 3 follow up appointments. For all 72 patients with isoniazid mono-resistance, no relapses have been recorded on NTBS.

**Conclusion:**

Navigating conflicting national and international guidelines is challenging to manage at a local level. Here we present successful treatment outcomes in a cohort of patients treated without fluoroquinolones, or with regimens that have been amended to include them based on local expert judgement. We acknowledge limitations of loss to follow up and incomplete medical records.

**Disclosures:**

All Authors: No reported disclosures

